# Homology-Dependent Silencing by an Exogenous Sequence in the *Drosophila* Germline

**DOI:** 10.1534/g3.111.001925

**Published:** 2012-03-01

**Authors:** Maria Pöyhönen, Augustin de Vanssay, Valérie Delmarre, Catherine Hermant, Anne Laure Todeschini, Laure Teysset, Stéphane Ronsseray

**Affiliations:** Laboratoire Biologie du Développement, UMR7622, CNRS–Université Pierre et Marie Curie, 9 quai Saint-Bernard, 75005 Paris, France

**Keywords:** RNA silencing, epigenetics, germline, transposable elements, *Drosophila*

## Abstract

The study of *P* transposable element repression in *Drosophila melanogaster* led to the discovery of the *trans*-silencing effect (TSE), a homology-dependent repression mechanism by which a *P*-transgene inserted in subtelomeric heterochromatin (Telomeric Associated Sequences) represses in *trans*, in the female germline, a homologous *P-lacZ* transgene inserted in euchromatin. TSE shows variegation in ovaries and displays a maternal effect as well as epigenetic transmission through meiosis. In addition, TSE is highly sensitive to mutations affecting heterochromatin components (including HP1) and the Piwi-interacting RNA silencing pathway (piRNA), a homology-dependent silencing mechanism that functions in the germline. TSE appears thus to involve the piRNA-based silencing proposed to play a major role in *P* repression. Under this hypothesis, TSE may also be established when homology between the telomeric and target loci involves sequences other than *P* elements, including sequences exogenous to the *D. melanogaster* genome. We have tested whether TSE can be induced *via lacZ* sequence homology. We generated a *piggyBac-otu-lacZ* transgene in which *lacZ* is under the control of the germline *ovarian tumor* promoter, resulting in strong expression in nurse cells and the oocyte. We show that all *piggyBac-otu-lacZ* transgene insertions are strongly repressed by maternally inherited telomeric *P-lacZ* transgenes. This repression shows variegation between egg chambers when it is incomplete and presents a maternal effect, two of the signatures of TSE. Finally, this repression is sensitive to mutations affecting *aubergine*, a key player of the piRNA pathway. These data show that TSE can occur when silencer and target loci share solely a sequence exogenous to the *D. melanogaster* genome. This functionally supports the hypothesis that TSE represents a general repression mechanism which can be co-opted by new transposable elements to regulate their activity after a transfer to the *D. melanogaster* genome.

Transposable elements (TEs) are present in all organisms, and their activity can both induce severe deleterious effects by disrupting gene activity and create genetic novelties possibly useful from an evolutionary point of view (Wicker *et al.* 2007). Various mechanisms exist for repressing TE mobility, including autorepression by proteins encoded by TEs themselves and host defense mechanisms via DNA methylation, heterochromatin formation, and small RNA silencing (Cam *et al.* 2008; Girard and Hannon 2008; Slotkin and Martienssen 2007). In a given organism, these mechanisms can vary depending on the cellular context. For example in *Drosophila melanogaster*, TEs are regulated by different RNA silencing pathways in somatic and germline tissues (Dufourt *et al.* 2011; Hartig and Forstemann 2011; Li *et al.* 2009; Malone *et al.* 2009). In species that have been recently invaded by a particular family of TEs, it is possible to recover strains with or without these TEs. These strains are useful to study the mechanisms of repression since TEs containing strains can be crossed to control strains (devoid of the TEs) to genetically isolate and identify regulatory TE copies. *D. melanogaster* has been invaded in the last century by three families of TEs: the *I* factor, the *hobo* element, and the *P* element (Anxolabehere *et al.* 1988; Blackman *et al.* 1987; Blackman 1989; Chambeyron and Bucheton 2005; Engels 1989; Finnegan 1989; Rio 2002). The first one transposes via a RNA intermediate (Class I element), and the two others transpose via a DNA intermediate (Class II elements). These TEs can induce hybrid dysgenesis, a syndrome of genetic abnormalities (*e.g.*, high mutation rate, chromosomal breakages, sterility) which occurs in the germline of progeny produced by crossing females lacking these elements and males carrying theses elements (Blackman *et al.* 1987; Kidwell *et al.* 1977; Picard *et al.* 1978).

The *P* element presents a maternally inherited repression termed “P cytotype” (Engels 1979). P cytotype shows epigenetic transmission through meiosis because memory of this maternal effect can be detected for more than five generations (Coen *et al.* 1994; Engels 1979). Genetic investigations to identify *P* copies responsible for the establishment of P cytotype allowed the discovery that *P* elements inserted at the telomere of the *X* chromosome have very strong repressive capacities (Ronsseray *et al.* 1991, 1996; Stuart *et al.* 2002) that show the complex rules of epigenetic transmission over generations typical of the P cytotype (Coen *et al.* 1994; Niemi *et al.* 2004; Ronsseray *et al.* 1991). These elements are inserted in subtelomeric heterochromatin (Ronsseray *et al.* 1996; Stuart *et al.* 2002), *i.e.*, tandemly repeated noncoding sequences called “Telomeric Associated Sequences” (TAS) (Karpen and Spradling 1992). Repression elicited by these elements requires a certain length of common sequence between regulatory elements inserted in TAS and repressed elements located in euchromatin (Marin *et al.* 2000). It is sensitive to mutations affecting heterochromatin protein 1 (Ronsseray *et al.* 1996), a major component of heterochromatin, and to *aubergine* (Reiss *et al.* 2004; Simmons *et al.* 2007), a gene playing a major role in the small RNA-silencing pathway termed Piwi-interacting RNA (piRNA) silencing (Brennecke *et al.* 2007). Furthermore, *P* element−derived piRNAs have been found in ovaries of P strain females, which can be correlated to maternal effect of P cytotype (Brennecke *et al.* 2008).

The discovery of a transgenic system mimicking the P cytotype properties provided an important opportunity to analyze phenotypic, genetic, and molecular properties of *P* element repression established by telomeric *P* elements. A *P-lacZ* transgene carrying the *Escherichia coli β-galactosidase* gene in frame with sequence encoding the N terminal domain of the *P* element transposase that was inserted in the TAS of the *X* chromosome was shown to repress ovarian expression of second *P-lacZ* located on another chromosome: this phenomenon was termed *trans*-silencing effect (TSE) (Roche and Rio 1998; Ronsseray *et al.* 2003). TSE has become a key tool to study the underlying mechanism of P cytotype, allowing visualization of the distribution of repression in ovaries and even within ovarioles using simple histochemical X-gal staining (Ronsseray *et al.* 2003). Further studies showed that TSE (1) can be also established by *P*-transgenes inserted in the TAS of autosomal telomeres (Josse *et al.* 2008; Roche and Rio 1998); (2) is restricted to the germline (Josse *et al.* 2008); (3) shows variegation in ovaries when repression is incomplete (Josse *et al.* 2007); (4) has a maternal effect whose memory can persist for more than five generations (Josse *et al.* 2007); (5) involves both a chromosomally and a cytoplasmically transmitted factor (Josse *et al.* 2007); (6) is sensitive to mutants affecting HP1 and the piRNA pathway (Josse *et al.* 2007; Todeschini *et al.* 2010); and (7) is linked to maternal transmission of small RNAs derived from the telomeric transgenes (Todeschini *et al.* 2010), which were recently characterized as piRNAs (Muerdter *et al.* 2012).

TSE variegation results in a bimodal stochastic distribution of egg chamber staining, some showing very strong *lacZ* repression while others showing null repression. Intermediate staining is rarely observed. Inside a given egg chamber, the 15 nurse cells show, in most of the cases, identical *on* or *off* staining. It must be emphasized that TSE functions only in germline cells, the tissue in which *P* transposition is restricted (Laski *et al.* 1986), and does not function in ovarian somatic follicle cells (Josse *et al.* 2008). TSE therefore likely involves a germline-specific piRNA repression pathway. Because the piRNA-silencing pathway has been shown to affect a large number of different families of TEs, one of the remaining questions is whether TSE is specific to *P* element sequences or, on the contrary, whether TSE can be obtained between a telomeric and an euchromatic locus sharing sequence homology other than *P* element sequences.

We have functionally tested the latter possibility by constructing a transgene in which the *lacZ* sequence is carried by a TE different from the *P* element, that is, *piggyBac* (Cary *et al.* 1989; Fraser *et al.* 1995), which shares no sequence similarity with the *P* element. *LacZ* expression was placed under the control of a female germline promoter (*ovarian tumor*). *D. melanogaster* embryos were transformed by the *piggyBac-otu-lacZ* transgene, allowing recovery of several insertions strongly expressed in nurse cells and the oocyte, the two populations of germline cells of ovaries. In this article, we report that telomeric *P*-transgenes carrying *lacZ* strongly repress, in the germline, expression of a *lacZ* gene carried by *piggyBac* transgene insertions. This repression presents the genetic and phenotypic properties of TSE and is sensitive to *aubergine* mutants. *Trans*-silencing can therefore be established via solely *lacZ* homology. This shows that TSE can be established for sequences exogenous to the *D. melanogaster* genome. In addition, this strongly reinforces the hypothesis that TSE represents a typical homology-dependent piRNA repression mechanism in the germline and that its complex *trans*-generational epigenetic properties therefore reflect those of a piRNA pathway functioning in germline cells of ovaries.

## MATERIALS AND METHODS

### Establishment of the transgenic lines

**Figure 1:**
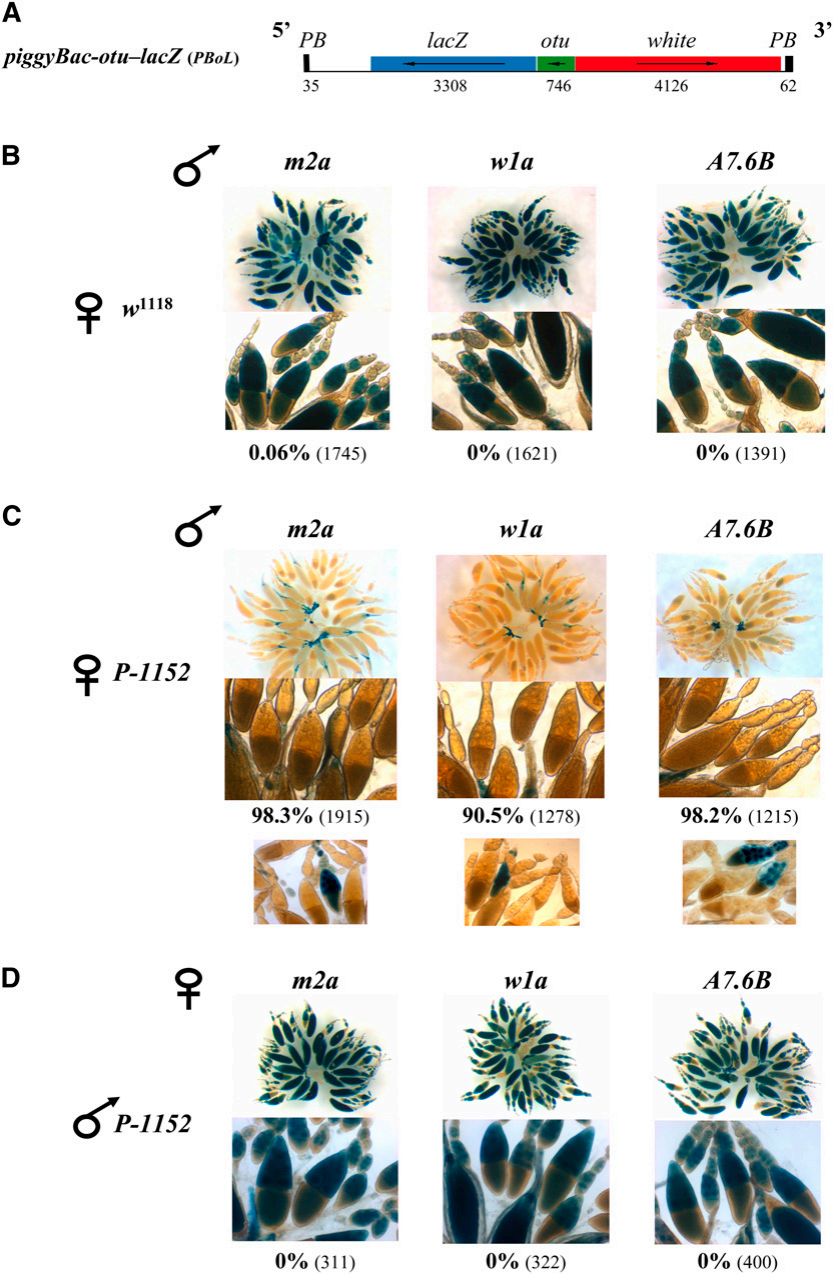
A telomeric *P-lacZ* strongly represses a *PBoL* transgene in the germline with a maternal effect. (A) Schematic representation of a *piggyBac-otu-lacZ* transgene named “*PBoL*”. *LacZ* expression is under the control of the *otu* promoter, and the *white* gene serves as a transformation marker. The length (bp) is indicated for the fragments constituting the transgene. *piggyBac* transformation vector sequences present at extremities of the transgene are in black: black boxes correspond to terminal regions of *piggyBac* (PB) and black lines to sequences required for germline transformation in *Drosophila*. (B-D) Three *PBoL* insertions (*m2a*, *w1a*, and *A7.6B*) were analyzed. Overnight lacZ staining of ovaries of G_1_ females produced by crossing males and females of the strains indicated above and to the left of the images are shown. The images were taken at lower (top) and greater (bottom) magnifications. In each case, the percentage of repressed egg chambers (at ovarian stages 9−10; see *Materials and Methods*) is given together with the total number of egg chambers counted between parenthesis. (B) The three *PBoL* insertions are strongly expressed in the germline (nurse cells and oocytes) due to the *otu* promoter. (C) These insertions are strongly repressed by a maternally inherited telomeric *P-lacZ* transgene (*P-1152*). Below the percentages, examples of variegating *lacZ* repression are shown. (D) *PBoL* insertions are not repressed by a paternally inherited *P-1152* locus.

The *piggyBac-otu-lacZ* transgene plasmid was generated by extracting two fragments, one containing the *white* gene and the second containing the *lacZ* gene under the control of the *ovarian tumor (otu*) gene promoter, from the *PCO* plasmids described in Boivin *et al.* 2003. These two fragments were cloned between the *Hin*dIII and *Eco*RI sites of *pXL-BacII* (Li *et al.* 2001; 2005). The transgene is 9815 bp long and is shown in Figure 1A. Transgenic lines were obtained by microinjection in the *w^1118^* strain (devoid of *P* elements) performed by the *BestGene* company. New insertions were further produced by remobilization of a primary insertion using the *jumpstarter* element encoding *piggyBac* transposase (Horn *et al.* 2003).

### Characterization of the transgenic lines

The transgenic lines were named “*PBoL*,” for *PiggyBac*-based transgenes containing the *lacZ* gene under control of the *otu* gene promoter. They carry the mini-*white* gene as transformation marker. Three *PBoL*-carrying lines were analyzed: *m2a*, *w1a*, and *A7.6B*.

The *m2a* and *w1a* insertions are located on chromosome *2*, and the *A7.6B* insertion is located on chromosome *3*. The *m2a* and *A7.6B* insertions are homozygous viable, whereas *w1a* is lethal and maintained over a balancer chromosome (*Cy*).

Precise localization of *PBoL* insertions was performed using inverse polymerase chain reaction (PCR) and the following oligonucleotides as primers: i2PCR3′Pb^-^ (GTTCCTTGTGTAGATGCATCTC), i2PCR5′Pb^+^ (GTCATTTTGACTCACGCGGTCG), i2PCR5′Pb^−^ (CGACCGCGTGAGTCAAAATGAC), iPCR5′Pb^+^ (ACTGAGATGTCCTAAATGCACAGC), iPCR3′Pb^−^ (GGATTTCACTGGAACTAGAATTCG), and iPCR5′Pb^+^ (ACTGAGATGTCCTAAATGCACAGC).

All lines apparently carry a single *PBoL* transgene insertion. The *m2a* insertion is located between the *mir-8* and the *Ugt37c1* genes at cytological site 53D. The *w1a* insertion is located in the *Arc-p20* gene at cytological site 26B. The *A7.6B* insertion is located in the *Alhambra* gene at cytological site 84B.

### *P*-element−derived transgenes and *Drosophila* lines

#### P-lacZ *fusion enhancer trap transgenes:*

*P-1152*, *P-1155* are enhancer-trap transgenes and contain an in-frame translational fusion of the *E. coli lacZ* gene to the second exon of the *P-transposase* gene. They carry *rosy*+ as a transformation marker (*P{lArB}* transgene) (O’Kane and Gehring 1987). *P-1152* (FBti0005700) comes from the stock previously known as #11152 in the *Bloomington Stock Center* and was mapped to the telomere of the *X* chromosome (site 1A); this stock carries two *P-lacZ* insertions in the same TAS unit and in the same orientation (Josse *et al.* 2007). *P-1155* (FBti0005691) comes from the stock previously known as #11155 of the *Bloomington Stock Center*. It contains a single *P-lacZ* insertion in TAS at the *3R* chromosome arm telomere (site 100F). *P-1152* and *P-1155* are homozygous viable and fertile. *P-1152* shows no *lacZ* expression in the ovary, whereas *P-1155* shows weak and nonuniform *lacZ* staining in follicle cells but no staining in the germline. The *T-1* line carries a cluster of *P-lacZ-white* elements (*P{lacW}* transgene) located at cytological site 50C on the second chromosome (Dorer and Henikoff 1994, 1997). The cluster contains seven transgene copies, including a defective copy, all inserted in direct orientation. In addition, the *T-1* line has complex chromosomal rearrangements, including translocations between the second and the third chromosomes due to X-ray treatment. After overnight staining, weak *lacZ* expression is detected in follicle cells of *T-1* ovaries, presumably because of a position effect at 50C, but no staining is observed in the germline. *P-1152*, *P-1155*, and *T-1* have a strong capacity to induce TSE which is maternally inherited (Josse *et al.* 2008; Roche and Rio 1998; Ronsseray *et al.* 2001).

Three strong mutant alleles of *aubergine* induced by EMS were used. All of them are homozygous female sterile. *aub^QC42^* (Schupbach and Wieschaus 1991) comes from the Bloomington Stock Center (stock #4968) and has not been characterized at the molecular level. *aub^HN2^* (Schupbach and Wieschaus 1991) has an amino acid substitution. *aub^N11^* (Wilson *et al.* 1996) has a 154-bp deletion, resulting in a frameshift that is predicted to add 16 novel amino acids after residue 740 (Harris and MacDonald 2001).

All stocks used carrying transgenic insertions have a M genetic background (devoid of *P* transposable elements), as do the multimarked balancer stocks and those carrying *aubergine* mutations. The Canton^y^ and *w*^1118^ lines were used as control lines, and these are completely devoid of any *P* element or *P* element−derived transgene (M lines) and of any *piggyBac*-derived transgene.

### Experimental conditions

All crosses were performed at 25° and involved three to five couples in most of the cases. Ovary *lacZ* expression assays were performed using X-gal overnight staining as described in Josse *et al.* 2007, except experiments involving *aubergine* mutants for which 24-hr staining was conducted because weaker *lacZ* expression required these conditions to facilitate scoring of TSE.

### Quantification of TSE

When TSE is incomplete, variegation is observed because “*on*/*off*” *lacZ* expression occurs between egg chambers (Josse *et al.* 2007). TSE was quantified by determining the percentage of egg chambers with no expression among ovarian stages 9-10 because *lacZ* expression of *PBoL* insertions was intense and reproducible at these stages.

## RESULTS

### Production of *PBoL* transgenic lines

A transgene was designed to test whether “non *P*-element” homology between a telomeric transgene and a target euchromatic transgene allows *trans*-silencing to take place. More precisely, we asked whether a telomeric *P-lacZ* transgene can repress, in *trans*, a transgene carrying the *lacZ* sequence in a TE other than the *P* element. The *piggyBac*-based transgenic system was used. The *Trichoplusia ni piggyBac* element is absent from the *Drosophila melanogaster* genome and shows no significant sequence similarity with the *P* element, as tested by BLAST analysis (data not shown). Because TSE is restricted to the female germline, expression of the *lacZ* gene in *piggyBac* was placed under the control of the germline-specific promoter of the *otu* (Figure 1A). After transformation of embryos and remobilization, six transgenic lines were recovered (named *PBoL*). All insertions but one showed β-galactosidase expression restricted to germline cells of the ovary. However, *lacZ* expression levels varied from one *PBoL* insertion to another, likely because of position effects. The three lines showing the strongest *lacZ* expression were selected for further analysis. Details about these lines are given in *Materials and Methods*. These lines are called *m2a* and *w1a* (chromosome *2*) and *A7.6B* (chromosome *3*). For all *PBoL* insertions, *lacZ* expression in ovaries was assayed in two different genetic backgrounds (Canton*^y^* and *w*^1118^) to take into account possible background effects on transgene expression. No significant difference was observed between the two backgrounds (data not shown). The *m2a*, *w1a*, and *A7.6B* insertions produced strong *lacZ* expression in nurse cells, especially at late stages of oogenesis and in mature oocytes (Figure 1B). Scoring egg chambers at stages 9-10 allowed detection of *lacZ* expression in all (but one) egg chamber among more than 4700 egg chambers assayed for the three *PBoL* insertions tested (Figure 1B).

### *LacZ* homology between a telomeric and an euchromatic locus allows *trans*-silencing to take place in germline cells of the ovary

*P-lacZ* insertions located in subtelomeric heterochromatin (TAS) of the *X* chromosome induce strong repression of any *P-lacZ* transgene inserted in euchromatin expressed in the female germline (Josse *et al.* 2008; Roche and Rio 1998; Ronsseray *et al.* 2001). In addition, incomplete repression results in variegation for X-gal staining from one egg chamber to another (Josse *et al.* 2007). This repression shows a strong maternal effect because strong repression is observed only when the telomeric locus is maternally inherited (Josse *et al.* 2008; Ronsseray *et al.* 2001). For example, crossing *P-1152* females with males carrying an euchromatic *P-lacZ* transgene resulted in G_1_ females showing 80% to 95% of egg chambers with repressed *lacZ* expression, whereas the reciprocal cross resulted in only 15% to 30% repression in G_1_ females (Josse *et al.* 2007, 2008). When *P-1152* females were crossed with males carrying any one of the three *PBoL* insertions tested, G_1_ females showed strong *lacZ* silencing in all cases (Figure 1C: 98% for *m2a* and *A7.6B* ; 90% for *w1a*). In addition, in each case, incomplete repression resulted in variegating *lacZ* expression characterized by on/off egg chamber *lacZ* expression (Figure 1C). Finally, the reciprocal cross was performed, and no repression was detected with any of the three *PBoL* insertions tested (Figure 1D). Therefore, *lacZ* homology allows *trans*-silencing to take place in the female germline and repression shows phenotypic and genetic properties of TSE.

### *PBoL* repression by autosomal silencers

Previous studies of TSE allowed the identification of several silencers located on autosomes (Josse *et al.* 2008; Roche and Rio 1998). First, *P-lacZ* transgenes inserted in subtelomeric heterochromatin of chromosomes *2* and *3* were found to be able to establish strong repression of a *P-lacZ* target transgene (Josse *et al.* 2008). This repression is also maternally inherited and shows variegation: for example, the *P-1155* telomeric *P-lacZ* transgene, located in the TAS of the *3R* chromosomal arm, was shown to repress a *P-lacZ* transgene located in euchromatin of chromosome *3* (named *P-Co1*). This repression is however weaker (TSE = 65%) than that induced by *X* chromosome telomeric insertion *P-1152* [TSE = 88% (Josse *et al.* 2008)]. Second, complete *trans*-silencing of *P-lacZ* was found to be induced by the *T-1* line (Ronsseray *et al.* 2001), which carries a cluster of *P-lacZ* transgenes (Dorer and Henikoff 1994, 1997) inducing local heterochromatin formation (Fanti *et al.* 1998) and which has complex chromosomal rearrangements induced by X-rays. Again *trans*-silencing was maternally inherited (Ronsseray *et al.* 2001).

The capacity of these two silencer loci to repress *PBoL* insertions was tested. The *P-1155* telomeric transgene induced repression of the three *PBoL* insertions tested (Table 1). *P-1155*-mediated repression is weaker than that obtained for *P-1152* (Figure 1) with *m2a* (56% *vs.* 98%) and *w1a* (81% *vs.* 90%), whereas strong repression was observed for both *P-1155* and *P-1152* with *A7.6B* (93% and 98%). Table 1 also shows that *T-1* induced complete silencing of the three *PBoL* tested. Note that in this case, repression can result from both *lacZ* and *white* homology. Autosomal *P-lacZ* silencers can thus strongly repress *PBoL* transgenes. In addition, a maternal effect was found for both the *P-1155* and *T-1* autosomal silencers because no repression was observed in the progeny of reciprocal crosses (Table 1).

**Table 1:** *PBoL* transgenes are repressed by autosomal TSE silencers

Line name	*m2a*	*w1a*	*A7.6B*
*Canton^y^*			
♀ \♂	0% (1344)	0% (1077)	0% (1457)
♂\♀	0% (420)	0% (429)	0% (368)
*P-1155*			
♀\ ♂	56.3% (448)	81.2% (399)	93.2% (382)
♂\♀	0% (409)	0.3% (344)	0% (305)
*T-1*			
♀ \♂	100% (769)	100% (465)	100% (544)
♂\♀	0% (332)	0% (303)	0% (353)

Reciprocal crosses were performed between individuals indicated in column 1 and in line 1. TSE was measured in G_1_ females. In each case, the first line (*♀\ ♂*) gives the percentage of TSE (total number of egg chambers scored in parenthesis) in progeny produced by crossing females indicated in column 1 with males indicated in line 1. The second line (*♂ \♀*) gives the percentage of TSE observed in progeny of the reciprocal cross (total number of egg chambers scored in parenthesis). *P-1155* carries a *P-lacZ-rosy* transgene, similar to *P-1152*, located at the telomere of the *3R* chromosomal arm. The *T-1* line carries a tandem array of seven *P-lacZ-white* transgenes located in the middle of the *2R* chromosomal arm. The Canton***^y^*** reference line is devoid of any *P*-transgene or *P*-element. *PBol*, *PiggyBac*-based transgenes containing the *lacZ* gene under control of the *otu* gene promoter; TSE, *trans*-silencing effect.

Further, we tested whether single transgenes located in euchromatin and heterochromatin (pericentromeric heterochromatin and fourth chromosome) can repress *PBoL* insertions. Indeed, previously such transgenes were shown to be unable to repress a *P-lacZ* transgene expressed in the female germline (Josse *et al.* 2008). Similarly, of five euchromatic *P-lacZ* insertions, and three pericentromeric chromosome insertions tested, located on chromosomes *X*, *2* and *3*, none repressed *m2a*, *w1a* or *A7.6B* (Table S1). In addition, a *P-lacZ* transgene located on the heterochromatic fourth chromosome, previously shown to be unable to repress a *P-lacZ* transgene (Josse *et al.* 2008), did not repress the *PBoL* insertions. Therefore, *P-lacZ* and *PBoL* target transgenes respond in the same way (repressed or not repressed) to all silencer/nonsilencer loci tested.

### A telomeric *P-lacZ* locus can repress two *PBoL* transgenes inserted on different chromosomes

A single telomeric *P-1152* locus was previously shown to be able to strongly repress two *P-lacZ* targets located at allelic or nonallelic positions (Josse *et al.* 2008). We tested whether a single *P-1152* locus can similarly repress two *PBoL* insertions. Females having maternally inherited *P-1152* and carrying two *PBoL* insertions presented more than 80% of repressed egg chambers (Table 2). This result was obtained for flies homozygous for the *m2a* insertion or for flies carrying two nonallelic *PBoL* insertions located on the same or on different chromosomes (*m2a* and *w1a* located on chromosome *2* and *A7.6B* located on chromosome *3*). Therefore, a single *P-1152* locus can repress simultaneously two *PBoL* insertions located at allelic or nonallelic positions. In addition, we found that a single maternally inherited *P-1152* locus established strong *lacZ* repression in females carrying both a hemizygous *P-lacZ* insertion located on chromosome *3* [*BQ16* (Josse *et al.* 2007)] and a hemizygous *PBoL* insertion (*m2a*; 93.8% of TSE, n = 241).

**Table 2:** A telomeric *P-lacZ* locus can repress two *PBoL* transgenes inserted at allelic or nonallelic positions

Row	Parental Cross	Genotype of G_1_ Females Analyzed	% TSE	n
1	♀ *m2a* x ♂ *m2a*	*+* / *+* ; *m2a* / *m2a* ; *+* / *+*	0.0	381
2	♀ *m2a* x ♂ *w1a*	*+* / *+* ; *m2a* / *w1a* ; *+* / *+*	0.0	844
3	♀ *m2a* x ♂ *A7.6B*	*+* / *+* ; *m2a* / *+* ; *+* / *A7.6B*	0.0	316
4	♀ *P-1152* ; *m2a* x ♂ *P-1152* ; *m2a*	*P-1152* / *P-1152* ; *m2a* / *m2a* ; *+* / *+*	95.8	406
5	♀ *P-1152* ; *m2a* x ♂ *w*^1118^	*P-1152* / *+* ; *m2a* / *+* ; *+* / *+*	84.2	310
6	♀ *P-1152* ; *m2a* x ♂ *m2a*	*P-1152* / *+* ; *m2a* / *m2a* ; *+* / *+*	87.3	512
7	♀ *P-1152* ; *m2a* x ♂ *w1a*	*P-1152* / *+* ; *m2a* / *w1a* ; *+* / *+*	90.2	877
8	♀ *P-1152* ; *m2a* x ♂ *A7.6B*	*P-1152* / *+* ; *m2a* / *+* ; *+* / *A7.6B*	97.4	381

The parental crosses shown in column 2 were performed in order to generate G_1_ females whose genotype is given in column 3. In each case, G_0_ females carrying *P-1152* were homozygous for this locus. LacZ staining of G_1_ female ovaries was performed and TSE was measured. Columns 4 and 5 give the TSE percentage and the total number of egg chambers counted, respectively.

### *LacZ* homology−dependent silencing is sensitive to mutations affecting the piRNA pathway gene *aubergine*

TSE has been shown to be highly sensitive to mutations affecting the piRNA silencing pathway (Josse *et al.* 2007; Todeschini *et al.* 2010). Repression of a *PBoL* target transgene by a *P-lacZ* telomeric locus was tested in *aubergine* mutant contexts (Table 3). *aubergine* is one of the main actors involved in the piRNA amplification mechanism termed “ping-pong” which functions in the germline (Brennecke *et al.* 2007; Li *et al.* 2009; Malone *et al.* 2009). TSE between *P-lacZ* transgenes was found previously to be null in *aubergine* heteroallelic mutant contexts (Josse *et al.* 2007). Similarly, *P-1152* repression of the *PBoL A7.6B* insertion was almost completely (2.6%) or completely abolished in the two *aubergine* heteroallelic mutant contexts tested (Table 3). Therefore *lacZ* homology-based *trans*-silencing is dependent on *aubergine*.

**Table 3:** *PBoL trans*-silencing is sensitive to *aubergine* mutations

	*♀*	*P-1152aub^QC42^* +	*♀*	*P-1152aub^HN2^* +
		*P-1152* *Cy* +		*P-1152* *Cy* +
		P-1152aub^*^ +		P-1152aub^*^ +
		+ *Cy A7.6B*		+ *Cy A7.6B*
**♂** + *aub^N11^A7.6B*		**74.8%** (1483)		**60.2%** (1255)
*¬ Cy A7.6B*		*P-1152aub^QC42^* +		*P-1152aub^HN2^* +
		+* aub^N11^ A7.6B*		+ *aub^N11^ A7.6B*
		**2.3%** (1049)		**0.0%** (524)

Crosses between females (first line) and males (first column) of the indicated genotypes were performed. LacZ staining was carried out on ovaries of G_1_ females whose genotype is shown inside the table. The percentage of TSE is given for each progeny with the total number of egg chambers scored indicated in parenthesis. aub^*^ indicates either the maternal or paternal *aub* mutant allele, which cannot be discriminated. TSE, *trans*-silencing effect.

## DISCUSSION

The genome of natural populations of *Drosophila melanogaster* has been invaded by three TE families during the last century: *I*, *hobo*, and *P* (Blackman 1989; Engels 1989; Finnegan 1989). For all three, a repression mechanism was established, and for two of them, *P* and *I*, repression has been shown to involve a maternal effect and complex epigenetic transmission over generations (Bucheton *et al.* 1976; Engels 1979; Picard *et al.* 1978). *P* and *I* repression involves regulatory copies of these TEs located on all chromosomes (Engels 1979; Picard *et al.* 1978), but some master regulatory sites corresponding to copies inserted in heterochromatin have been identified (Jensen *et al.* 2002; Picard 1978; Ronsseray *et al.* 1991). Both *P* and *I* repression mechanisms are sensitive to mutations affecting heterochromatin formation and RNA silencing (Bucheton *et al.* 2002; Chambeyron *et al.* 2008; Klenov *et al.* 2007; Reiss *et al.* 2004; Ronsseray *et al.* 1996). For *P* element repression (P cytotype), the existence of a maternally transmitted cytoplasmic component (pre-P cytotype), coupled with chromosomally inherited *P* copies, was shown to be necessary to establish strong repression in the zygote (Niemi *et al.* 2004; Ronsseray *et al.* 1993). However, the maternally inherited component is not an autoreproducible component (Ronsseray *et al.* 1993; Sved 1987). Furthermore, upon discovery of the piRNA silencing pathway (Brennecke *et al.* 2007; Gunawardane *et al.* 2007) sequence analysis of piRNAs suggested that a high proportion of TEs are repressed in the gonads by this homology-dependent silencing mechanism. In particular, deep-sequencing of ovarian small RNAs allowed detection of piRNAs derived from *I* and *P* elements whose maternal transmission is correlated with repression of hybrid dysgenesis induced by massive transposition of these TEs (Brennecke *et al.* 2008). In the case of *P*, these maternally transmitted small RNAs very likely correspond to the pre-P cytotype.

All these data suggest a model in which the genome harbors several “traps” for invasive mobile DNA sequences which constitutively produce piRNAs and allow repression by a homology-dependent silencing mechanism. Some families (*gypsy*, *ZAM* and *Idefix*) are regulated by a heterochromatic locus called *flam-COM* located close to the centromere of the *X* chromosome (Desset *et al.* 2003; Mevel-Ninio *et al.* 2007; Pelisson *et al.* 1994; Prud’homme *et al.* 1995). This repression takes place in the somatic follicle cells of the ovary and, therefore, is mediated by a functionally different piRNA pathway (Desset *et al.* 2008; Malone *et al.* 2009; Pelisson *et al.* 2002). It is noteworthy that this regulation presents different genetic properties because *gypsy* repression, for example, does not exhibit a maternal effect nor *trans*-generational epigenetic transmission (A. Pélisson, personal communication). For the *I* factor, repression appears to involve homology-dependent silencing in the germline (Jensen *et al.* 1999, 2002; Malinsky *et al.* 2000; Robin *et al.* 2003) and major repressive loci appear to be located in pericentromeric heterochromatin (Jensen *et al.* 2002) and in the intercalary heterochromatin 42AB locus which contains numerous fragments of TEs (Brennecke *et al.* 2008). For the *P* element, *Telomeric-Associated Sequences* appear to be a major trap. Indeed, numerous P strains deriving from natural populations having various geographical origins have been found to carry *P* elements located at the telomere of the *X* chromosome (Ajioka and Eanes 1989; Ronsseray *et al.* 1989). Telomeric *P* elements inserted in TAS deriving from seven natural populations have been further isolated in a genomic background devoid of other *P* copies and were shown to have repressive capacities (Marin *et al.* 2000; Ronsseray *et al.* 1996, 1998; Stuart *et al.* 2002). The combination of a telomeric defective *P* element with various target *P*-transgenes showed that repression induced by the telomeric *P*-element is dependent on *P*-element homology between the telomeric and target loci (Marin *et al.* 2000; Roche and Rio 1998).

To validate and generalize this model, it remained, however, important to determine via a functional assay whether *trans*-silencing can be established via sequence homology other than that derived from the *P* element. In the present article, we show that the *lacZ* gene located inside a *piggyBac*-derived transgene is strongly repressed by a maternally inherited telomeric *P-lacZ* transgene, this repression exhibiting variegation, which is a typical phenotype of TSE. The parallel between *P-lacZ* and *PBoL* repression by a telomeric *P-lacZ* also includes the capacity to be repressed by autosomal silencers and sensitivity to *aubergine* mutations. Therefore, this functional assay indicates that repression by sequences inserted in TAS is not a *P*-element restricted property but rather a more general repression system that may function for other TEs inserted in TAS, including those recently introduced in the genome. Thus, TSE not only allows us to address the nature of P cytotype but also corresponds to a sensitive or appropriate tool to investigate phenotypic and genetic properties of a piRNA silencing pathway functioning in nurse cells.

Taking into account the epigenetic *trans*-generational effects of TSE and its variegating phenotype (Josse *et al.* 2007), predictive assumptions can be proposed for properties of a germline-specific piRNA pathway. For example, variegation between egg chambers inside ovaries resembles position effect variegation in the eye observed for genes located close to heterochromatin (Girton and Johansen 2008), and this suggests that target repression by piRNAs may involve heterochromatin formation. In addition, long-term memory of the maternal effect observed over generations with TSE (Josse *et al.* 2007) indicates that piRNA-based repression functioning in the germline undergoes amplification that can transcend a single fly generation to reach its maximum level. Note that *P* is not the only TE displaying long-term inheritance of its repressive properties because *I* factor regulation also shows such *trans*-generational effects (Bucheton 1979; Jensen *et al.* 1999; Picard *et al.* 1978). It will be interesting to investigate whether such a long-term *trans*-meiosis epigenetic inheritance exists for other piRNA producing loci in the genome.

## Supplementary Material

Supporting Information
