# Supplementary material for: Homology-Dependent Silencing by an Exogenous Sequence in the Drosophila Germline
Source: G3 (Bethesda). 2012 Mar 1;2(3):331–8. doi: 10.1534/g3.111.001925 (PMC3291502; doi:10.1534/g3.111.001925)
Supplement: Supporting Information [file supp_2_3_331__index.html]

Supporting Information 

# Homology-Dependent Silencing by an Exogenous Sequence in the *Drosophila* Germline

## Supporting Information for Poyhonen *et al*, 2012

**Files in this Data Supplement:**

- Table S1 - Non‐telomeric single *P‐lacZ* transgenes do not repress *PBoL* transgenes (PDF, 63 KB)
