# Supplementary material for: Homology-Dependent Silencing by an Exogenous Sequence in the Drosophila Germline
Source: G3 (Bethesda). 2012 Mar 1;2(3):331–8. doi: 10.1534/g3.111.001925 (PMC3291502; doi:10.1534/g3.111.001925)
Supplement: Supporting Information [file supp_2.3.331_TableS1.pdf]

Table S1 Non-telomeric single *P-lacZ* transgenes do not repress *PBoL* transgenes.

Chromosome	Insertion name	Transgene structure	Cytological site	Flybase ID	*m2a*	*w1a*	*A7.6B*
**Euchromatic site**							
Chromosome *X*	***P-592****	*P{lArB}*	1E	FBti0005682	443	465	398
	P-1033	*P{lArB}*	35D-E	FBti0003389	304	-	432
Chromosome *2*	***P-1069***	*P{lArB}*	28E	FBti0004644	457	-	540
	P-1169	*P{lArB}*	65C-D	FBti0005696	361	326	610
Chromosome *3*	***P-1173***	*P{lArB}*	84E	FBti0005134	460	488	455
**Heterochromatic site**							
Chromosome *X* (pericentromeric)	***P-2004***	*P{lacW}*	20A-B (*h26-h32*)	FBti0013902	375	345	312
Chromosome *2* (pericentromeric)	***CH(2)6***	*P{PZ}*	2R (*h42-h44*)	FBti0003833	387	390	296
Chromosome *3* (pericentromeric)	***P-1784***	*P{PZ}*	80A-F	FBti0003533	604	336	365
Chromosome *4*	***P-6303****	*P{lacW}*	101F	FBti0002288	321	427	732

Single *P-lacZ* transgenes located in euchromatin and heterochromatin were tested for their capacity to induce silencing of *PBoL* insertions. Transgene information is given: chromosomal location (column 1), insertion name (column 2), structure (column 3), cytological location on larval polytene chromosomes (column 4), *Flybase* Identification number (column 5). * indicates that the insertion is maintained at the homozygous state. All these insertions are unable to repress a *P-lacZ* target transgene in the germline (Josse et al. 2008 and data not shown). TSE on *PBoL* targets was measured in G_1 females produced by crossing females carrying the insertion indicated in column 2 and *PBoL* males indicated at the top of columns 6-8 (*m2a, w1a*, and *A7.6B*): no silencing was observed (TSE=0%) irrespective of the *P-lacZ* and *PBoL* insertions tested. The total number of egg chambers scored for each cross is given in columns 6-8.
